# HLA-DRB1 and –DQB1 Alleles, Haplotypes and Genotypes in Emirati Patients with Type 1 Diabetes Underscores the Benefits of Evaluating Understudied Populations

**DOI:** 10.3389/fgene.2022.841879

**Published:** 2022-03-24

**Authors:** Zain Al Yafei, Steven J. Mack, Marion Alvares, Bassam R. Ali, Bachar Afandi, Salem A. Beshyah, Charu Sharma, Wael Osman, Rajaa Mirghani, Amre Nasr, Sareea Al Remithi, Jamal Al Jubeh, Wasim Y. Almawi, Juma AlKaabi, Gehad ElGhazali

**Affiliations:** ^1^ Sheikh Khalifa Medical City, Purehealth, Abu Dhabi, United Arab Emirates; ^2^ Department of Pediatrics, University of California, San Francisco, San Francisco, CA, United States; ^3^ Department of Genetics and Genomics, College of Medicine and Health Sciences, United Arab Emirates University, Al Ain, United Arab Emirates; ^4^ Department of Internal Medicine, Tawam Hospital, Al Ain, United Arab Emirates; ^5^ Department of Medicine, Dubai Medical College, Dubai, United Arab Emirates; ^6^ Department of Internal Medicine, United Arab Emirates University, Al Ain, United Arab Emirates; ^7^ College of Arts and Sciences, Khalifa University, Abu Dhabi, United Arab Emirates; ^8^ Higher College of Technology, Abu Dhabi, United Arab Emirates; ^9^ Department of Basic Medical Sciences, College of Medicine, King Saud Bin Abdulaziz University for Health Sciences, Riyadh, Saudi Arabia; ^10^ Department of Biomedical Sciences, Nazarbayev University School of Medicine, Astana, Kazakhstan

**Keywords:** type 1 diabetes, HLA, haplotypes, Emiratis, ethnicity

## Abstract

**Background:** HLA class II (DR and DQ) alleles and antigens have historically shown strong genetic predisposition to type 1 diabetes (T1D). This study evaluated the association of *DRB1* and *DQB1* alleles, genotypes, and haplotypes with T1D in United Arab Emirates.

**Materials and Methods:** Study subjects comprised 149 patients with T1D, and 147 normoglycemic control subjects. Cases and controls were Emiratis and were *HLA-DRB1* and *-DQB1* genotyped using sequence-based typing. Statistical analysis was performed using Bridging Immunogenomic Data-Analysis Workflow Gaps R package.

**Results:** In total, 15 *DRB1* and 9 *DQB1* alleles were identified in the study subjects, of which the association of DRB1*03:01, DRB1*04:02, DRB1*11:01, DRB1*16:02, and DQB1*02:01, DQB1*03:02, DQB1*03:01, and DQB1*06:01 with altered risk of T1D persisted after correcting for multiple comparisons. Two-locus haplotype analysis identified DRB1*03:01∼DQB1*02:01 [0.44 vs. 0.18, OR (95% CI) = 3.44 (2.33–5.1), *Pc* = 3.48 × 10^−10^]; DRB1*04:02∼DQB1*03:02 [0.077 vs. 0.014, OR = 6.06 (2.03–24.37), *Pc* = 2.3 × 10^−3^] and DRB1*04:05∼DQB1*03:02 [0.060 vs. 0.010, OR = 6.24 (1.79–33.34), *Pc* = 0.011] as positively associated, and DRB1*16:02∼DQB1*05:02 [0.024 vs. 0.075, OR = 0.3 (0.11–0.74), *Pc* = 0.041] as negatively associated with T1D, after applying Bonferroni correction. Furthermore, the highest T1D risk was observed for *DR3/DR4* [0.104 vs. 0.006, OR = 25.03 (8.23–97.2), *Pc* = 2.6 × 10^−10^], followed by *DR3/DR3* [0.094 vs. 0.010, OR = 8.72 (3.17–25.32), *Pc* = 3.18 × 10^−8^] diplotypes.

**Conclusion:** While *DRB1* and *DQB1* alleles and haplotypes associated with T1D in Emiratis showed similarities to Caucasian and non-Caucasian populations, several alleles and haplotypes associated with T1D in European, African, and Asian populations, were not observed. This underscores the contribution of ethnic diversity and possible diverse associations between *DRB1* and *DQB1* and T1D across different populations.

## Introduction

Type I diabetes (T1D) is a complex autoimmune disorder characterized by insulin deficiency resulting from autoimmune destruction of insulin-secreting pancreatic β-cells in genetically predisposed individuals (Eisenbarth, 2010).

The International Diabetes Federation estimates that more than 1.1 million individuals below the age of 20 years are diagnosed with T1D worldwide (International Diabetes Federation, 2019). In this regard, it was reported that the incidence of T1D in children (15 years or younger) varies greatly among different ethnic and racial groups, highlighted by high to intermediate incidence among Europeans e.g., 5.2/100,000 per year in Finland (International Diabetes Federation, 2019; Knip, 2021), and the low incidence among Asians e.g., 1.8/100,000 per year in China (Gong et al., 2015; International Diabetes Federation, 2019). Among Arab populations of the Middle East, it was estimated that there are about 60,000 children with T1D under the age 15 years (Zayed, 2016). Of these, Kuwait and Saudi Arabia rank among the top ten countries worldwide in T1D prevalence, with rates of 44.5/100,000 and 33.5/100,000, respectively (Majeed et al., 2014; Shaltout et al., 2017; International Diabetes Federation, 2019). No reliable data have yet been reported on the T1D incidence in the United Arab Emirates (UAE). While the reason underlying the differences in T1D prevalence rate according to ethnicity and geographical locations is not known (Patterson et al., 2014; IDF 2019), it was suggested that both environmental parameters and genetic influence the initiation of T1D development (Eisenbarth, 2010; Blanter et al., 2019; Xia et al., 2019).

More than 60 genetic loci were reported in the literature as associated with altered T1D susceptibility (Todd et al., 2007; Bergholdt et al., 2012; Xia et al., 2019), of which the Human Leukocyte Antigens (HLA) class II genes contributed to approximately one-half of T1D genetic risk (Noble et al., 1996; Pociot et al., 2010). Individual *DRB1* and *DQB1* alleles, or allelic combinations were associated with the altered risk of T1D, and both susceptible and protective DRB1, DQA1, and DQB1 alleles were reportedly implicated with T1D pathogenesis (Noble et al., 1996; Erlich et al., 2008). This was highlighted by the association of DRB1*03:01:01∼DQB1*02:01 and DRB1*04:01:01∼DQB1*03:02 haplotypes with increased susceptibility to T1D among Bahraini Arabs (Al-Jenaidi et al., 2005). As the frequencies of *HLA* alleles, haplotypes, and genotypes show considerable population and ethnic differences, population studies confirmed that the relationship of *HLA* with T1D also varies according to the geographical location and ethnic background (Al-Jenaidi et al., 2005; Ahmadov et al., 2018; Fawwad et al., 2019; Zabeen et al., 2019). Compared to numerous studies performed on European populations, limited numbers of studies have been conducted on populations in the Middle East-North Africa (MENA) region (Al-Jenaidi et al., 2005; Noble et al., 2013).

This is the first case-control study investigating the association of *HLA* with T1D in the Emirati population. The present-day UAE national population is characterized by high rates of consanguinity, endogamy, along with sizeable extended families, and tribal community nature (Zayed, 2016). These and other features further justify investigating the association of *HLA*-class II antigens in T1D among Emirati Arabs. In addition to screening individual alleles, we aimed to assess the prevalence of *DRB1∼DQB1* haplotype combination in search of specific susceptible and protective haplotypes.

## Materials and Methods

### Study Population

The study subjects comprised 149 unrelated **patients** with T1D and 147 unrelated normoglycemic controls who were recruited from outpatient T1D clinics at Sheikh Khalifa Medical City (Abu Dhabi, UAE) and Tawam Hospital (Al-Ain, UAE). T1D was diagnosed based on clinical features and laboratory findings and on the 1985 World Health Organization (WHO) criteria (WHO Technical Report Series 727; World Health Organization, 1985) and the classification of diabetes mellitus document, WHO, Geneva; 2019 (World Health Organization, 1985; World Health Organisation, 2019). The inclusion criteria were patients who presented with acute symptoms of diabetes (e.g., polyuria, polydipsia and weight loss), had random blood glucose >200 mg/dl (11.1 mmol/L), C-peptide levels of <0.3 mmol/L, and had required long term insulin therapy since the time of diagnosis. Exclusion criteria included monogenic diabetes recognized by autosomal dominant mode of inheritance, type 2 diabetes as manifested by obesity and signs of insulin resistance, diabetes diagnosed before the age of 12 months or chronic diseases (especially autoimmune diseases) and patients/guardians who were unable to provide consent to participate in the study. Control individuals consisted of university students and healthy children with normal fasting/random blood glucose levels, no family history of T1D or other autoimmune diseases and were matched for age and gender with patients with T1D. Healthy children (≤16 years) were enrolled during routine hospital visits for other complaints while medical students (adolescents/adults) were recruited from the Tawam teaching hospital (Al Ain, UAE). For each recruited patient with T1D, a control was matched for age and gender to the extend possible. The mean ± SD age were 18.32 ± 7.45 (range 6–26 years) and 19.71 ± 6.93 (6–24) of cases and controls, respectively. Patients with T1D and their controls were Emirati nationals with grandparents born in the United Arab Emirates. Table 1 presents basic clinical and demographic characteristics of the study population.

**TABLE 1:** Clinical and demographic characteristics of the study population.

Variable	Patients (149)	Control (147)
Age	18.32 ± 7.45 years	19.71 ± 6.93
Gender		
Male	69 (46.2)	65 (44.2)
Female	80 (53.8)	82 (55.8)
Mode of presentation for TID		
DKA	64 (42.9)	-
Hyperglycemia	68 (45.6)	
Duration of diabetes	10.51 ± 6.15	-
<5 years	33 (22.1)	-
5–10 years	42 (28.5)	-
>10 years	74 (49.7)	-
HbA1c (%)	8.6 ± 1.9	-
Established autoimmune diseases	23 (17.2)	None
Celiac disease	5 (3.6)	None
Thyroid disease	18 (12.1)	None
Family History of T1D	50 (33.6)	None
Family history of autoimmune diseases	62 (42)	None

This study was performed following the guidelines of the Declaration of Helsinki (1993). The study protocol was approved by the Institutional Review Boards at SKMC (REC-25-10-2016 RS-445) and United Arab Emirates University (AAMDHREC 2016-4255 16-002), and informed consent was acquired from all study subjects or their guardians before performing the study.

### Human Leukocyte Antigen Typing

All samples were analysed in our laboratory, which is a well-established clinical laboratory accredited by both the College of American Pathologists (CAP) and ISO15189. Total genomic DNA was extracted from EDTA-anticoagulated peripheral blood of study subjects using Qiagen DNA Mini kit on automated QIAcube, according to the manufacturer instructions (Qiagen, Hilden, Germany). DNA concentrations were determined using NanoDrop-2000 (Wilmington, DE, United States), and *HLA-DRB1* and -*DQB1* genotyping was performed by sequence-based typing (SBT), using GenDx AlleleSEQR kits (GenDx, https://www.gendx.com/, Netherland). Based on the method, exon 2 was sequenced for HLA-DRB1(AlleleSEQR HLA-DRB1, product code 08K63-03), and exon 2 and 3 were sequenced for HLA-DQB1 (AlleleSEQR HLA-DQBQ1, product code 08K64-03). All reagents necessary for primary amplification and sequencing were included in the HLA-DRB1 and –DQB1 AlleleSEQR SBT kits (GenDx, Netherland). Briefly, following the primary amplification using 9700 thermal cycler (Applied Biosystems, United States), the PCR products were purified by ExoSAP-IT and the PCR products were sequenced using BigDye Terminatory chemistry and the ABI-3130 genetic sequencer (Applied Biosystems, Foster City, CA). Finally, the sequence data were analysed using the GenDx SBTengine *HLA* typing software (http://www.gendx.com/product_line/sbtengine/), as described in the manufacturer’s protocol. Quality control measures consisted of randomly-chossing 10% of cases and control samples for retyping; concordance was 100%. In addition, six samples from the CAP External Quality Control Proficiency testing program were used to ensure control for the genotyping procedures.

### Statistical Analysis

The association of *HLA-DRB1* and -*DQB1* alleles and haplotypes with T1D was analysed using the Bridging Immunogenomic Data-Analysis Workflow Gaps (BIGDAWG) package (version 2.1) (Pappas et al., 2016), with 95% confidence intervals (CI), odds ratios (OR), and *p*-values (P) reported. BIGDAWG’s haplotype estimation function, which required the R “haplo.stats” package (version 1.7.7), was also used. Allele counts < 5 in study subjects were collapsed automatically through BIGDAWG into “binned” category (Pappas et al., 2016). The same analyses were performed at the haplotype level, which were estimated by the expectation-maximization (EM) approach (Dempster et al., 1977), and haplotypes with counts of three or fewer were binned. The corrected *p* values (Pc) for multiple comparisons were calculated using the Benferroni method. Analysis of Hardy-Weinberg equilibrium (HWE) deviations for specific haplotypes and individual loci was performed using PyPop (version 0.8.0), and Guo and Thompson’s exact method was used in identifying significant locus-level HWE deviations (Guo and Thompson, 1992; Lancaster et al., 2007). Individual haplotype deviations from HWE expectations were determined by Chen’s method (Guo and Thompson, 1992), at a significance of 0.05.

The pould (phased or unphased LD) R package (version 0.10.4.9000) (https://cran.r-project.org/package=pould) (Osoegawa et al., 2019) was used to calculate D′, *W*_*n*_, *W*_*a/b*_, and *W*_*b/a*_ global linkage disequilibrium (LD) values for *DRB1∼DQB1* haplotypes (Thomson and Single 2014), in the range of 0 (equilibrium) to 1 (linkage). In LD, D’ signifies the weighted average of normalized disequilibrium (D_ij_) values, and it is less sensitive to variation across haplotypes of polymorphic loci than the other measures. Moreover, *W*_*n*_ indicates a correlation coefficient describing the association between alleles at two loci. The conditional asymmetric LD (ALD) measures, including *W*_*a/b*_ and *W*_*b/a*_, extend the *W*_*n*_ measure for variation at one locus (a) conditioned on the variation at the second (b) (Single et al., 2016).

## Results

### Deviation From HWE

There was no deviation from HWE at *DRB1* and *DQB1* loci among controls, and no HWE deviation for *DRB1* locus in patients, however, the *DQB1* locus indicated an overall deviation from HWE (*p* = 0.0006) in patients. No trend toward excess heterozygosity or homozygosity was detected in patients. This deviation was due to the presence of five rare *DQB1* genotypes—DQB1*02:03+DQB1*03:04 [1 observed (obs); 0.0268 expected (exp); *p* = 0.0271], DQB1*03:03+DQB1*04:01 (1 obs; 0.0067 exp; *p* = 0.0073), DQB1*02:03+DQB1*06:03 (1 obs; 0.0403 exp; *p* = 0.0388), DQB1*06:03+DQB1*06:03 (1 obs 0.0151 exp; *p* = 0.0106), and DQB1*05:01+DQB1*06:09 (1 obs; 0.0469 exp; *p* = 0.0358). Many of these alleles are observed less than five times in patients or controls (Supplementary Tables S1, S3).

### HLA-DRB1 and -DQB1 Allele Frequencies

Significant DRB1 allelic differences were seen between T1D patients and control subjects, 10 of 31 alleles differing significantly (*p* < 0.05). When the Bonferroni correction was applied, differences were significant for only 4, which comprised DRB1*03:01 [0.44 vs. 0.187, OR (95% CI) = 3.46 (2.35–5.11), *Pc* = 3.2 × 10^−10^] and DRB1*04:02 [0.081 vs. 0.014, OR = 6.35 (2.14–25.44), *Pc* = 2.0 × 10^−3^], which were higher among patients, and DRB1*11:01 [0.003 vs. 0.044, OR = 0.07 (0–0.49), *Pc* = 0.015] and DRB1*16:02 [0.023 vs. 0.078, OR = 0.28 (0.1–0.7), *Pc* = 0.030], which were higher in control subjects (Table 2). Similarly, significant allelic differences were seen at the *DQB1* locus, 5 of 9 alleles differing significantly after the Bonferroni correction. These comprised DQB1*02:01 [0.48 vs. 0.19, OR = 3.78 (2.58–5.57), *Pc* = 3.04 × 10^−12^] and DQB1*03:02 [0.21 vs. 0.08, OR = 3.1 (1.82–5.40), *Pc* = 6.01 × 10^−5^] which were higher among patients, and DQB1*03:01 [0.02 vs. 0.11, OR = 0.16 (0.05–0.40), *Pc* = 5.64 × 10^−5^], DQB1*05:01 [0.05 vs. 0.11, OR = 0.40 (0.19–0.80), *Pc* = 0.044], and DQB1*06:01 [0.003 vs. 0.06, OR = 0.05 (0–0.33), *Pc* = 5.84 × 10^−4^], which were higher among control subjects (Table 3).

**TABLE 2:** Associations of *DRB1* alleles with T1D in Emirati study subjects.

Allele	Patient^a^	Control^a^	OR (95%CI)	*p*-value	*P*_*corrected*_ ^b^
01:01	4 (0.013)^c^	7 (0.024)	0.56 (0.12–2.22)	0.349	NA
01:02	5 (0.017)	8 (0.027)	0.61 (0.16–2.15)	0.386	NA
03:01	132 (0.44)	55 (0.187)	3.46 (2.35–5.11)	2.1 × 10^−11^	**3.2 × 10** ^**−10**^
04:02	24 (0.081)	4 (0.014)	6.35 (2.14–25.44)	1.3 × 10^−4^	**2.0 × 10** ^**−3**^
04:03	6 (0.02)	15 (0.051)	0.38 (0.12–1.06)	0.042	0.630
04:05	24 (0.081)	8 (0.027)	3.13 (1.33–8.19)	4.0 × 10^−3^	0.060
07:01	25 (0.084)	31 (0.105)	0.78 (0.43–1.4)	0.370	NA
10:01	6 (0.020)	13 (0.044)	0.44 (0.14–1.28)	0.096	NA
11:01	1 (0.003)	13 (0.044)	0.07 (0–0.49)	1.0 × 10^−3^	**0.015**
11:04	1 (0.003)	10 (0.034)	0.1 (0–0.68)	6.0 × 10^−3^	0.086
13:02	1 (0.003)	10 (0.034)	0.1 (0–0.68)	6.0 × 10^−3^	0.086
15:01	3 (0.01)	12 (0.041)	0.24 (0.04–0.9)	0.017	0.227
15:02	2 (0.007)	10 (0.034)	0.19 (0.02–0.91)	0.018	0.238
16:01	18 (0.060)	16 (0.054)	1.12 (0.53–2.39)	0.754	NA
16:02	7 (0.023)	23 (0.078)	0.28 (0.1–0.7)	2.0 × 10^−3^	**0.030**

a Study subjects comprised 149 T1D patients and 147 normoglycemic healthy controls. Significant differences are reported in bold.

b Pcorrected = corrected *p* value, calculated as P x n, where *n* = number of comparisons.

c Number (frequency).

**TABLE 3:** Associations of *DQB1* alleles with T1D in Emirati study subjects.

Allele	T1D patient^a^	Control^a^	OR (95%CI)	*p*-value	*P*_*corrected*_ ^b^
02:01	142 (0.48)^c^	57 (0.19)	3.78 (2.58–5.57)	3.38 × 10^−13^	**3.04 × 10** ^**−12**^
02:02	26 (0.09)	28 (0.10)	0.91 (0.5–1.65)	0.736	NA
03:01	6 (0.02)	33 (0.11)	0.16 (0.05–0.4)	6.27 × 10^−6^	**5.64 × 10** ^**−5**^
03:02	62 (0.21)	23 (0.08)	3.1 (1.82–5.4)	6.68 × 10^−6^	**6.01 × 10** ^**−5**^
04:02	2 (0.007)	12 (0.04)	0.16 (0.02–0.72)	6.00 × 10^−3^	0.053
05:01	14 (0.05)	32 (0.11)	0.4 (0.19–0.8)	5.00 × 10^−3^	**0.044**
05:02	28 (0.09)	46 (0.16)	0.56 (0.33–0.95)	0.021	0.174
06:01	1 (0.003)	18 (0.06)	0.05 (0–0.33)	6.49 × 10^−5^	**5.84 × 10** ^**−4**^
06:03	3 (0.01)	8 (0.03)	0.36 (0.06–1.54)	0.122	NA

a Study subjects comprised 149 T1D patients and 147 normoglycemic healthy controls. Significant differences are reported in bold.

b P_corrected_, = corrected *p* value, calculated as P x n, where n = number of comparisons.

c Number (frequency).

### Distribution of DRB1-DQB1 Haplotypes

In total, 95 distinct *DRB1∼DQB1* haplotypes were identified (Supplementary Table S2), of which 42 had counts of ≥2 in controls or patients, and thus were considered common. In total, four individual haplotypes showing a statistically significant association with T1D (Table 4). The full list of *DRB1∼DQB1* haplotypes that were binned due to low expected counts is given in Supplementary Table S4. Higher frequencies of DRB1*03:01∼DQB1*02:01 [0.44 vs. 0.18, OR = 3.44 (2.33–5.10), *Pc* = 3.48 × 10^−10^], DRB1*04:02∼DQB1*03:02 [0.077 vs. 0.014, OR = 6.06 (2.03–24.37), *Pc* = 2.3 × 10^−3^], and DRB1*04:05∼DQB1*03:02 [0.06 vs. 0.01, OR = 6.24 (1.79–33.34), *Pc =* 0.011] haplotypes, and lower frequency of DRB1*16:02∼DQB1*05:02 haplotype [0.024 vs. 0.075, OR (95% CI) = 0.30 (0.11–0.74), *Pc =* 0.041] were seen in T1D patients compared to controls. This assigned T1D susceptibility and protective nature to these haplotypes, respectively (Table 4).

**TABLE 4:** Association of *DRB1-DQB1* Haplotypes with T1D in Emirati study subjects.

Haplotype	T1D patient^a^	Controls^a^	OR (95%CI)	*p*-value	*P*_*corrected*_ ^b^
DRB1*01:01∼DQB1*05:01	4 (0.013)^c^	7 (0.024)	0.56 (0.12–2.22)	0.35	NA
DRB1*01:02∼DQB1*05:01	4 (0.013)	8 (0.027)	0.49 (0.11–1.84)	0.23	NA
DRB1*03:01∼DQB1*02:01	130 (0.44)	54 (0.18)	3.44 (2.33–5.1)	3.16 × 10^−11^	**3.48 × 10** ^**−10**^
DRB1*04:02∼DQB1*03:02	23 (0.077)	4 (0.014)	6.06 (2.03–24.37)	2.1 × 10^−4^	**2.3 × 10** ^**−3**^
DRB1*04:03∼DQB1*03:02	5 (0.017)	15 (0.051)	0.32 (0.09–0.94)	0.021	0.208
DRB1*04:05∼DQB1*03:02	18 (0.060)	3 (0.010)	6.24 (1.79–33.34)	9.6 × 10^−4^	**0.011**
DRB1*07:01∼DQB1*02:02	21 (0.071)	27 (0.092)	0.75 (0.39–1.42)	0.34	NA
DRB1*10:01∼DQB1*05:01	5 (0.017)	13 (0.044)	0.37 (0.1–1.12)	0.05	0.431
DRB1*11:01∼DQB1*03:01	1 (0.003)	10 (0.034)	0.1 (0–0.68)	5.7 × 10^−3^	0.061
DRB1*16:01∼DQB1*05:02	18 (0.060)	16 (0.054)	1.12 (0.53–2.39)	0.75	NA
DRB1*16:02∼DQB1*05:02	7 (0.024)	22 (0.075)	0.3 (0.11–0.74)	3.8 × 10^−3^	**0.041**

a Study subjects comprised 149 T1D patients and 147 normoglycemic healthy controls. Significant differences are reported in bold.

b Pcorrected, = corrected *p* value for multiple comparisons as per Bonferroni correction method.

c Number (frequency).

### Distribution of DRB1-DQB1 Diplotypes

Parallel to the differential distribution of 2-locus haplotypes among T1D patients and controls, distinct *DRB1∼DQB1 + DRB1∼DQB1* diplotypes were seen in T1D patients. Extensive diversity in the diplotypes identified was seen, and as such we focused on the DRB1*03- and DRB1*04-containing diplotypes for analysis. As shown in Table 5, DRB1*03:01∼DQB1*02:01 + DRB1*04:02/05∼DQB1*03:02 0.104 vs. 0.006, OR = 25.03 (8.23–97.20, *p* = 2.6 × 10^−10^) followed by the homozygous DRB1*03:01∼DQB1*02:01 + DRB1*03:01∼DQB1*02:01 [0.094 vs. 0.01, OR = 8.72 (3.17–25.32), *p* = 3.18 × 10^−8^] were associated with heightened risk for T1D.

**TABLE 5:** Associations of *DRB1*∼*DQB1* diplotypes with T1D in the study subjects.

Diplotype^a^	Patient^b^	Control^b^	OR (95% CI)	*p*-value
03:01–02:01/04:xx∼03:02^c^	31 (0.104)^d^	2 (0.006)	25.03 (8.23–97.2)	**2.6 x 10** ^**−10**^
03:01–02:01/03:01–02:01	28 (0.094)	3 (0.010)	8.72 (3.17–25.32)	**3.18 x 10** ^**−8**^
04:xx∼03:02/04:xx∼03:02	4 (0.013)	0 (0.000)	NA	NA

a DRB1∼DQB1/DRB1∼DQB1 diplotype.

b Study subjects consisted of 149 T1D subjects and 147 normoglycemic control subjects. Significant differences are reported in bold.

c xx = HLA-DRB1*04 alleles *04:02 and *04:05.

d Number (frequency).

### Linkage Disequilibrium Between DRB1 and DQB1 Loci

D′ and *W*_*n*_ values, as well as *W*_*DRB1/DQB1*_ and *W*_*DQB1/DRB1*_ measures*,* which assess the global linkage disequilibrium (LD) between *DRB1* and *DQB1* loci in T1D patients and control groups are shown in Supplementary Table S5. There were no substantial differences between T1D cases and controls in terms of LD. Strong LD between *DRB1* and *DQB1* loci is illustrated by the high D’ measure (0.92 in patients and 0.95 in controls), and to a lesser extent by *W*_*n*_ (0.75 in patients and 0.72 in controls). The ALD measures (*W*_*DRB1/DQB1*_ and *W*_*DQB1/DRB*_) dissect the variation on each locus conditioned on the other. Comparison of the W_*DRB1/DQB1*_ (0.89 in patients and 0.90 in controls) with *W*_*DQB1/DRB1*_ (0.71 in both groups) indicated less variation among *DRB1* alleles related to *DQB1*, when compared to *DQB1* alleles related to *DRB1*.

## Discussion

The HLA contribution to T1D genetic susceptibility differs between populations and ethnic groups (Noble et al., 2013), largely due to varied frequencies and functional associations of *DRB1* and *DQB1* alleles and haplotypes (susceptible, protective). Key *DRB1* and *DQB1* alleles and haplotypes associated with T1D were reported for Caucasian and non-Caucasian populations (Petrone et al., 2001; Erlich et al., 2008; Ilonen et al., 2009; Noble et al., 2013), which included DRB1*03:01, DRB1*04:02, DRB1*04:05, DQB1*02:01, and DQB1*03:02 along with DRB1*03:01∼DQB1*02:01 (DR3), DRB1*04:02∼DQB1*03:02 (DR4), and DRB1*04:05∼DQB1*03:02 haplotypes also observed in this study and studies on other Arab populations from Bahrain, Tunisia, Lebanon and Saudi Arabia (Al-Harbi et al., 2004; Al-Jenaidi et al., 2005; Stayoussef et al., 2009; Manan et al., 2010; El-Amir et al., 2015; Eltayeb-Elsheikh et al., 2020). It is noteworthy that no DR3 or DR4 haplotype linked with T1D was detected in East and Southeast Asians (Hashimoto et al., 1994; Kawabata et al., 2002).

DRB1*04:01∼DQB1*03:02 (0.34% controls frequency) and DRB1*04:01∼DQB1*03:01 (not seen in controls) were rare in Emiratis. This was reminiscent of findings in Europeans where DRB1*04:01∼DQB1*03:02 haplotype the second susceptibility haplotype, compared to DRB1*04:01∼DQB1*03:01 which was protective in Europeans (Erlich et al., 2008; Noble et alel., 2013), but not Africans (Onengut-Gumuscu et al., 2019). In our study, the DRB1*04:03 allele and DRB1*04:03∼DQB1*03:02 haplotype were more prevalent in the healthy controls but were not statistically different after correcting for multiple comparisons. The highest T1D risk in Emiratis was conferred by DRB1*04:05∼DQB1*03:02, DRB1*04:02∼DQB1*03:02 and DRB1*03:01∼DQB1*02:01 haplotypes. By comparison, the highest risk in European was imparted by DRB1*04:05∼DQB1*03:02, DRB1*04:01∼DQB1*03:02, DRB1*04:02∼DQB1*03:02, and DRB1*03:01∼DQB1*02:01 (Erlich et al., 2008).

DQB1*03:03, DRB1*08:01, DRB1*08:02, and DRB1*09:01 were rare among patients or controls in the present study. Moreover, DRB1*03:02 was rare in the Emirati study population (Al-Yafei et al., 2019; Arnaiz-Villena et al., 2019) and European derived populations; and frequent in DQB1*04:02-containing haplotypes in Africans, protecting against T1D (Howson et al., 2013). DQB1*04:01, common in East Asians (Kawabata et al., 2002), is rare in Emiratis (Al-Yafei et al., 2019; Arnaiz-Villena et al., 2019). Furthermore, several *DRB1∼DQB1* haplotypes associated with T1D in Europeans, Asians, and Africans were not observed here. For example, the DRB1*04:05∼DQB1*02:01 haplotype predisposed to T1D susceptibility in African Americans, while the African DR3 (DRB1*03:02∼DQB1*04:02) haplotype was protective (Noble et al., 2013). In addition to DR3 (DRB1*03:01∼DQB1*02:01) and DR4 (DRB1*04∼DQB1*03:02, DRB1*08:01∼DQB1*04:02 is a T1D predisposing haplotype in Europeans (Thomson et al., 2007). The African DRB1*03:02∼DQB1*04:02 and DRB1*04:05∼DQB1*02:01 haplotypes were rare in our study and in European populations. The DQB1*04:02 allele was more prevalent in the Emirati healthy controls but was not statistically different after correcting for multiple comparisons. DRB1*xx:xx ∼ DQB1*04:02 haplotypes were detected at low counts. The DRB1*08:02∼DQB1*03:02, DRB1*09:01∼DQB1*03:03, and DRB1*04:05∼DQB1*04:01 haplotypes, associated with increased T1D susceptibility in East Asians (Ikegami et al., 2008; Katahira et al., 2009), were rare in the current study population (Supplementary Tables S2, S4). The DRB1*04:05∼DQB1*04:01 haplotype common in East Asia populations (Japanese, Taiwanese, Philippines), is rare in Europeans and African Americans (Thomson et al., 2007), in line with the findings here. On the other hand, DRB1*09:01∼DQB1*03:03, also rare in African Americans, is found in Europeans and East Asians (Thomson et al., 2007). It should be noted that a unique (DR9) DRB1*09:01∼DQA1*03:01∼DQB1*02:02 haplotype (but not the DRB1*09:01∼DQB1*03:03) haplotype has been detected in Africans (Noble et al., 2013).

While high prevalence of DRB1*07:01∼DQB1*02:02 was seen in controls (9.18%) and cases (7.05%), no significant difference in its distribution between cases and controls was seen. DRB1*07:01∼DQA1*02:01∼DQB1*02:02 conferred some protection against T1D in Europeans (Erlich et al., 2008), while DRB1*07:01∼DQA1*03:01∼DQB1*02:02 was susceptible for T1D in Africans (Noble et al., 2013). Varied T1D risk of *DR7*-containing haplotypes was dependent on the *DQA1* allele contained in the DRB1*07:01∼DQA1*xx:xx∼DQB1*02:02 haplotype. Noble et al. (2013) showed that the DRB1*07:01∼DQB1*03:03 haplotype was protective of T1D in Europeans but was rare in Africans; this haplotype is rare in the current study (0.34% in both groups). Moreover, the distribution of DRB1*01:01∼DQB1*05:01, DRB1*01:02∼DQB1*05:02, DRB1*16:01∼DQB1*05:02, and DRB1*10:01∼DQB1*05:01 haplotypes were comparable between T1D cases and controls. While DRB1*01:01/01:02∼DQB1*05:01 was shown earlier to be associated with increased susceptibility to T1D (Thomson et al., 2007), this was not consistent with subsequent studies (Erlich et al., 2008). The DRB1*16:02∼DQB1*05:02 haplotype was protective for T1D while DRB1*11:01∼DQB1*03:01 and DRB1*10:01∼DQB1*05:01 were more prevalent in the healthy controls but were not statistically different after correcting for multiple comparisons. DRB1*15:01∼DQB1*06:02, DRB1*14:01∼DQB*05:03, and DRB1*07:01∼DQB1*03:03 (Noble et al., 1996; Petrone et al., 2001; Noble et al., 2011) are protective in Europeans but were observed at low frequencies in the present study and were binned (Supplemental S2 and S4). DRB1*15:01-DQB1*06:02 was seen in approximately 20% of Europeans, but in only 1% of T1D individuals (Noble et al., 1996). DRB1*16:02-DQB1*05:02 is common in controls (7.48%) and patients (2.35%), but is rare in Europeans, Africans, and Asians (Hashimoto et al., 1994). DRB1*16:01∼DQB1*05:02, a common DR2 haplotype in the studied population (6.04 and 5.44% in patients and controls, respectively), was not associated with T1D. In addition, DRB1*15:01∼DQB1*06:02, a T1D protective haplotype in most ethnicities (Osoegawa et al., 2019), was detected at low frequency in patients (0.67%) and controls (1.67%). Moreover, the (protective) DRB1*15:03∼DQB1*06:02, DRB1*08:04∼DQB1*03:01, and DRB1*03:02∼DQB1*04:02 African haplotypes were rare in our study population.

Differential contribution of *DR∼DQ* haplotypes to T1D risk were recognized; this was dictated by the specific ethnic background. Most of our T1D patients carried (DRB1*03:01∼DQB1*02:01) (*DR3*) or (DRB1*04:02/05∼DQB1*03:02) (*DR4*) haplotypes (87%), and 23% of these were carriers of the heterozygous *DR3*/*DR4* diplotype. In particular, the DRB1*03:01∼DQB1*02:01/DRB1*04:02/05∼DQB1*03:02 (heterozygous) diplotype imparted the highest T1D risk, followed by DRB1*03:01∼DQB1*02:01/DRB1*03:01∼DQB1*02:01 (homozygous) diplotype. This was comparable to Europeans, where most (90%) T1D patients were *DR3* or *DR4* carriers, with 40% carrying the DR3/DR4 diplotype (Ronningen et al., 1991; Noble et al., 1996), which is higher than the 12% *DR3*/*DR4* diplotype carrier rate seen in Africans (Noble et al., 1996). Furthermore, the highest risk for T1D in Europeans was conferred by the *DR3*/*DR4* diplotype, rather than *DR3/DR3* (Noble et al., 1996; Hermann et al., 2003; Erlich et al., 2008), while in East Asians, the highest T1D risk was conferred by the *DR4*/*DR9* diplotype (Ikegami et al., 2008). This absence of *DR3* might explain the low prevalence of T1D in these populations, as suggested earlier (Ikegami et al., 2008).

The apparent discrepancies between our results and those of other populations can be attributed to several factors, including race/ethnicity, incidence of T1D, sample size, genotyping methods, and other factors (Park, 2007; Polychronakos and Li 2011). This was highlighted by the *HLA* allele/haplotype frequencies between our population and Europeans, Africans, and East Asians (Al-Yafei et al., 2019; Arnaiz-Villena et al., 2019). This in turn explains, at least in part, the variations in T1D prevalence and incidence (Patterson et al., 2012). The high LD values between *DRB1* and *DQB1* loci were found in Emiratis, consistent with the findings in other ethnic groups. LD values were similar in cases and controls, and were identical for *W*_*DQB1/DRB1*_, suggesting equivalent diversity of *DQB1* alleles within *DRB1∼DQB1* haplotypes between the groups. The intermediate *W*_*n*_ LD measure value relative to *W*_*DRB1/DQB1*_ and *W*_*DQB1/DRB1*_ measures in patients illustrates the importance for applying the ALD measures for highly polymorphic genetic systems.

## Conclusion

The highest T1D risk was imparted by the DRB1*03:01∼DQB1*02:01/DRB1*04:02-05∼DQB1*03:02 diplotype followed by the DRB1*03:01∼DQB1*02:01/DRB1*03:01∼DQB1*02:01 diplotype. Emiratis showed similarities with and differences from European, Asian, and African populations in terms of *HLA-DRB1* and -*DQB1* alleles and haplotypes and their associations with T1D. However, our study had some shortcomings, namely the relatively small sample size, and lack of *DQA1* genotyping (especially for *DR7*+ samples). An interesting dimension of ethnic diversity and possible diverse relationships is highlighted between *HLA*-*DRB1* and -*DQB1* genes and T1D across different populations.

## Data Availability

All sequences were submitted to the Short Read Archive (SRA) at GenBank and assigned BioSample accession numbers between SAMN26674334 and SAMN26674604 under BioProject PRJNA609073.
