# Supplementary material for: HLA-DRB1 and –DQB1 Alleles, Haplotypes and Genotypes in Emirati Patients with Type 1 Diabetes Underscores the Benefits of Evaluating Understudied Populations
Source: Front Genet. 2022 Mar 24;13:841879. doi: 10.3389/fgene.2022.841879 (PMC8997289; doi:10.3389/fgene.2022.841879)
Supplement: Supplementary file 1 [file DataSheet1.docx]

**Supplementary Material Table S1**

| **Supplementary Table S1: Frequency of *DRB1 and DQB1* alleles in patients with T1D and controls** | | | |
| --- | --- | --- | --- |
| **Locus** | **Allele** | **Patients**  **N (%)** | **Controls**  **N (%)** |
| *DRB1* | 01:01 | 4 (1.3) | 7 (2.4) |
| *DRB1* | 01:02 | 5 (1.7) | 8 (2.7) |
| ***DRB1*** | **03:01** | **132 (44.3)** | **55 (18.7)** |
| *DRB1* | 03:02 | 0 | 2 (0.7) |
| *DRB1* | 03:05 | 7 (2.3) | 0 |
| *DRB1* | 04:01 | 6 (2.0) | 2 (0.7) |
| ***DRB1*** | **04:02** | **24 (8.1)** | **4 (1.4)** |
| ***DRB1*** | **04:03** | **6 (2.0)** | **15(5.1)** |
| *DRB1* | 04:04 | 5 (1.7) | 1 (0.3) |
| ***DRB1*** | **04:05** | **24 (8.1)** | **8 (2.7)** |
| *DRB1* | 04:06 | 2 (0.7) | 7 (2.4) |
| *DRB1* | 04:07 | 1 (0.3) | 2 (0.7) |
| *DRB1* | 04:08 | 3 (1.0) | 0 |
| *DRB1* | 04:10 | 1 (0.3) | 0 |
| *DRB1* | 04:13 | 3 (1.0) | 0 |
| *DRB1* | 04:41 | 0 | 1 (0.3) |
| ***DRB1*** | **07:01** | **25 (8.4)** | **31 (10.5)** |
| *DRB1* | 08:01 | 0 | 1 (0.3) |
| *DRB1* | 08:02 | 0 | 1 (0.3) |
| *DRB1* | 08:04 | 3 (1.0) | 2 (0.7) |
| *DRB1* | 09:01 | 5 (1.7) | 0 |
| ***DRB1*** | **10:01** | **6 (2.0)** | **13 (4.4)** |
| *DRB1* | 11:01 | 1 (0.3) | 13 (4.4) |
| *DRB1* | 11:02 | 1 (0.3) | 4 (1.4) |
| *DRB1* | 11:03 | 0 | 1 (0.3) |
| *DRB1* | 11:04 | 1 (0.3) | 10 (3.4) |
| *DRB1* | 11:10 | 0 | 1 (0.3) |
| *DRB1* | 12:01 | 0 | 3 (1.0) |
| *DRB1* | 12:02 | 0 | 3 (1.0) |
| *DRB1* | 12:10 | 0 | 1 (0.3) |
| *DRB1* | 13:01 | 1 (0.3) | 7 (2.4) |
| *DRB1* | 13:02 | 1 (0.3) | 10 (3.4) |
| *DRB1* | 13:03 | 0 | 3 (1.0) |
| *DRB1* | 14:01 | 0 | 1 (0.3) |
| *DRB1* | 14:02 | 0 | 1 (0.3) |
| *DRB1* | 14:04 | 1 (0.3) | 6 (2.0) |
| *DRB1* | 14:54 | 0 | 1 (0.3) |
| *DRB1* | 15:01 | 3 (1.0) | 12 (4.1) |
| *DRB1* | 15:02 | 2 (0.7) | 10 (3.4) |
| *DRB1* | 15:03 | 0 | 3 (1.0) |
| *DRB1* | 15:06 | 0 | 2 (0.7) |
| *DRB1* | 15:11 | 0 | 1 (0.3) |
| ***DRB1*** | **16:01** | **18 (6.0)** | **16 (5.4)** |
| ***DRB1*** | **16:02** | **7 (2.3)** | **23 (7.8)** |
| *DRB1* | 16:09 | 0 | 1 (0.3) |
| *DRB1* | 16:10 | 0 | 1 (0.3) |
| ***DQB1*** | **02:01** | **142 (47.7)** | **57 (19.4)** |
| ***DQB1*** | **02:02** | **26 (8.7)** | **28 (9.5)** |
| *DQB1* | 02:03 | 4 (1.3) | 3 (1.0) |
| *DQB1* | 02:05 | 0 | 1 (0.3) |
| *DQB1* | 03:01 | 6 (2.0) | 33 (11.2) |
| ***DQB1*** | **03:02** | 62 (20.8) | 23 (7.8) |
| *DQB1* | 03:03 | 2 (0.7) | 3 (1.0) |
| *DQB1* | 03:04 | 2 (0.7) | 2 (0.7) |
| *DQB1* | 03:05 | 1 (0.3) | 0 |
| *DQB1* | 03:19 | 0 | 1 (0.3) |
| *DQB1* | 03:49 | 0 | 1 (0.3) |
| *DQB1* | 04:01 | 1 (0.3) | 1 (0.3) |
| *DQB1* | 04:02 | 2 (0.7) | 12 (4.1) |
| ***DQB1*** | **05:01** | **14 (4.7)** | **32 (10.9)** |
| ***DQB1*** | **05:02** | **28 (9.4)** | **46 (15.6)** |
| *DQB1* | 05:03 | 1 (0.3) | 9 (3.1) |
| *DQB1* | 06:01 | 1 (0.3) | 18 (6.1) |
| *DQB1* | 06:02 | 2 (0.7) | 6 (2.0) |
| *DQB1* | 06:03 | 3 (1.0) | 8 (2.7) |
| *DQB1* | 06:04 | 0 | 6 (2.0) |
| *DQB1* | 06:05 | 0 | 1 (0.3) |
| *DQB1* | 06:08 | 0 | 1 (0.3) |
| *DQB1* | 06:09 | 1 (0.3) | 2 (0.7) |

Entries for DRB1 and DQB1 alleles, with expected counts > 5 in patients or controls are indicated in **boldface**.

Other entries “binned alleles”.

**Supplementary Material Table S2**

| **Supplementary Table S2: Frequency of *DRB1~DQB1* haplotypes in patients with T1D and controls** | | |
| --- | --- | --- |
| **DRB1~DQB1** | **Patients**  **N (%)** | **Control**  **N (%)** |
| **01:01~05:01** | **4 (1.34)** | **7 (2.38)** |
| 01:02~03:02 | 1 (0.34) | 0 |
| **01:02~05:01** | **4 (1.34)** | **8 (2.72)** |
| **03:01~02:01** | **130 (43.62)** | **54 (18.37)** |
| 03:01~02:02 | 0 | 1 (0.34) |
| 03:01~02:03 | 2 (0.67) | 0.00% |
| 03:02~04:02 | 0 | 2 (0.68) |
| 03:05~02:01 | 2 (0.67) | 0 |
| 03:05~02:02 | 3 (1.01) | 0 |
| 03:05~05:02 | 2 (0.67) | 0 |
| 04:01~02:01 | 1 (0.34) | 0 |
| 04:01~03:01 | 1 (0.34) | 0 |
| 04:01~03:02 | 4 (1.34) | 1 (0.34) |
| 04:01~03:49 | 0 | 1 (0.34) |
| 04:02~02:01 | 1 (0.34) | 0.00% |
| **04:02~03:02** | **23 (7.72)** | **4 (1.36)** |
| **04:03~03:02** | **5 (1.68)** | **16 (5.10)** |
| 04:03~03:05 | 1 (0.34) | 0 |
| 04:04~02:01 | 2 (0.67) | 0 |
| 04:04~03:02 | 3 (1.01) | 0 |
| 04:04~04:02 | 0 | 1 (0.34) |
| 04:05~02:01 | 1 (0.34) | 0.00% |
| 04:05~02:02 | 2 (0.67) | 0 |
| 04:05~02:03 | 2 (0.67) | 3 (1.02) |
| **04:05~03:02** | **18 (6.04)** | **3 (1.02)** |
| 04:05~04:01 | 1 (0.34) | 1 (0.34) |
| 04:05~05:02 | 0 | 1 (0.34) |
| 04:06~04:02 | 2 (0.67) | 7 (2.38) |
| 04:07~03:02 | 1 (0.34) | 0 |
| 04:07~03:03 | 0 | 1 (0.34) |
| 04:07~05:03 | 0 | 1 (0.34) |
| 04:08~03:01 | 2 (0.67) | 0 |
| 04:08~03:04 | 1 (0.34) | 0 |
| 04:10~03:02 | 1 (0.34) | 0 |
| 04:13~03:02 | 3 (1.01) | 0 |
| 04:41~04:02 | 0 | 1 (0.34) |
| 07:01~02:01 | 3 (1.01) | 2 (0.68) |
| **07:01~02:02** | **21 (7.05)** | **27 (9.18)** |
| 07:01~02:05 | 0 | 1 (0.34) |
| 07:01~03:03 | 1 (0.34) | 1 (0.34) |
| 08:01~04:02 | 0 | 1 (0.34) |
| 08:02~03:01 | 0 | 1 (0.34) |
| 08:04~03:01 | 1 (0.34) | 2 (0.68) |
| 08:04~03:04 | 1 (0.34) | 0 |
| 08:04~05:02 | 1 (0.34) | 0 |
| 09:01~02:01 | 1 (0.34) | 0 |
| 09:01~03:02 | 3 (1.01) | 0 |
| 09:01~03:03 | 1 (0.34) | 0 |
| 10:01~02:01 | 1 (0.34) | 0 |
| **10:01~05:01** | **5 (1.68)** | **13 (4.42)** |
| **11:01~03:01** | **1 (0.34)** | **10 (3.40)** |
| 11:01~03:04 | 0 | 1 (0.34) |
| 11:01~06:01 | 0 | 1 (0.34) |
| 11:01~06:03 | 0 | 1 (0.34) |
| 11:02~02:01 | 0 | 1 (0.34) |
| 11:02~03:01 | 0 | 2 (0.68) |
| 11:02~03:19 | 0 | 1 (0.34) |
| 11:02~06:03 | 1 (0.34) | 0 |
| 11:03~03:01 | 0 | 1 (0.34) |
| 11:04~03:01 | 1 (0.34) | 9 (3.06) |
| 11:04~06:03 | 0 | 1 (0.34) |
| 11:10~03:04 | 0 | 1 (0.34) |
| 12:01~03:01 | 0 | 1 (0.34) |
| 12:01~05:01 | 0 | 2 (0.68) |
| 12:02~03:01 | 0 | 3 (1.02) |
| 12:10~03:01 | 0 | 1 (0.34) |
| 13:01~03:03 | 0 | 1 (0.34) |
| 13:01~05:01 | 0 | 1 (0.34) |
| 13:01~06:03 | 1 (0.34) | 4 (1.36) |
| 13:01~06:08 | 0 | 1 (0.34) |
| 13:02~06:03 | 0 | 1 (0.34) |
| 13:02~06:04 | 0 | 6 (2.04) |
| 13:02~06:05 | 0 | 1 (0.34) |
| 13:02~06:09 | 1 (0.34) | 2 (0.68) |
| 13:03~03:01 | 0 | 2 (0.68) |
| 13:03~06:01 | 0 | 1 (0.34) |
| 14:01~05:03 | 0 | 1 (0.34) |
| 14:02~03:01 | 0 | 1 (0.34) |
| 14:04~05:03 | 1 (0.34) | 6 (2.04) |
| 14:54~05:03 | 0 | 1 (0.34) |
| 15:01~05:02 | 0 | 3 (1.02) |
| 15:01~06:01 | 0 | 5 (1.70) |
| 15:01~06:02 | 2 (0.67) | 4 (1.36) |
| 15:01~06:03 | 1 (0.34) | 0 |
| 15:02~05:01 | 1 (0.34) | 1 (0.34) |
| **15:02~06:01** | **1 (0.34)** | **9 (3.06)** |
| 15:03~06:02 | 0 | 2 (0.68) |
| 15:03~06:03 | 0 | 1 (0.34) |
| 15:06~05:02 | 0 | 2 (0.68) |
| 15:11~06:01 | 0 | 1 (0.34) |
| **16:01~05:02** | **18 (6.04)** | **16 (5.44)** |
| **16:02~05:02** | **7 (2.35)** | **22 (7.48)** |
| 16:02~06:01 | 0 | 1 (0.34) |
| 16:09~05:02 | 0 | 1 (0.34) |
| 16:10~05:02 | 0 | 1 (0.34) |

Entries for DRB1-DQB1 haplotypes with expected counts > 3 in patients or controls are indicated in **boldface**.

Other entries “binned haplotypes”.

**Supplementary Material Table S3**

| **Supplementary Table S3: Binned *DRB1* and *DQB1* alleles, with expected counts** < **5 in patients or controls** | | | |
| --- | --- | --- | --- |
| **Locus** | **Allele** | **Case** | **Control** |
| *DRB1* | 03:02 | 0 | 2 |
| *DRB1* | 03:05 | 7 | 0 |
| *DRB1* | 04:01 | 6 | 2 |
| *DRB1* | 04:04 | 5 | 1 |
| *DRB1* | 04:06 | 2 | 7 |
| *DRB1* | 04:07 | 1 | 2 |
| *DRB1* | 04:08 | 3 | 0 |
| *DRB1* | 04:10 | 1 | 0 |
| *DRB1* | 04:13 | 3 | 0 |
| *DRB1* | 04:41 | 0 | 1 |
| *DRB1* | 08:01 | 0 | 1 |
| *DRB1* | 08:02 | 0 | 1 |
| *DRB1* | 08:04 | 3 | 2 |
| *DRB1* | 09:01 | 5 | 0 |
| *DRB1* | 11:02 | 1 | 4 |
| *DRB1* | 11:03 | 0 | 1 |
| *DRB1* | 11:10 | 0 | 1 |
| *DRB1* | 12:01 | 0 | 3 |
| *DRB1* | 12:02 | 0 | 3 |
| *DRB1* | 12:10 | 0 | 1 |
| *DRB1* | 13:03 | 0 | 3 |
| *DRB1* | 14:01 | 0 | 1 |
| *DRB1* | 14:02 | 0 | 1 |
| *DRB1* | 14:04 | 1 | 6 |
| *DRB1* | 14:54 | 0 | 1 |
| *DRB1* | 15:03 | 0 | 3 |
| *DRB1* | 15:06 | 0 | 2 |
| *DRB1* | 15:11 | 0 | 1 |
| *DRB1* | 16:09 | 0 | 1 |
| *DRB1* | 16:10 | 0 | 1 |
| *DQB1* | 02:03 | 4 | 3 |
| *DQB1* | 02:05 | 0 | 1 |
| *DQB1* | 03:03 | 2 | 3 |
| *DQB1* | 03:04 | 2 | 2 |
| *DQB1* | 03:05 | 1 | 0 |
| *DQB1* | 03:19 | 0 | 1 |
| *DQB1* | 03:49 | 0 | 1 |
| *DQB1* | 04:01 | 1 | 1 |
| *DQB1* | 05:03 | 1 | 9 |
| *DQB1* | 06:04 | 0 | 6 |
| *DQB1* | 06:02 | 2 | 6 |
| *DQB1* | 06:05 | 0 | 1 |
| *DQB1* | 06:08 | 0 | 1 |
| *DQB1* | 06:09 | 1 | 2 |

**Supplementary Material Table S4**

| **Supplementary Table S4: Binned *DRB1****~****DQB1* haplotypes, with expected counts** < **3 in patients or controls** | | |
| --- | --- | --- |
| **DRB1~DQB1** | **patient** | **Control** |
| 01:02~03:02 | 1 | 0 |
| 03:01~02:02 | 0 | 1 |
| 03:01~02:03 | 2 | 0 |
| 03:02~04:02 | 0 | 2 |
| 03:05~02:01 | 2 | 0 |
| 03:05~02:02 | 3 | 0 |
| 03:05~05:02 | 2 | 0 |
| 04:01~02:01 | 1 | 0 |
| 04:01~03:01 | 1 | 0 |
| 04:01~03:02 | 4 | 1 |
| 04:01~03:49 | 0 | 1 |
| 04:02~02:01 | 1 | 0 |
| 04:03~03:05 | 1 | 0 |
| 04:04~02:01 | 2 | 0 |
| 04:04~03:02 | 3 | 0 |
| 04:04~04:02 | 0 | 1 |
| 04:05~02:01 | 1 | 0 |
| 04:05~02:02 | 2 | 0 |
| 04:05~02:03 | 2 | 3 |
| 04:05~04:01 | 1 | 1 |
| 04:05~05:02 | 0 | 1 |
| 04:06~04:02 | 2 | 7 |
| 04:07~03:02 | 1 | 0 |
| 04:07~03:03 | 0 | 1 |
| 04:07~05:03 | 0 | 1 |
| 04:08~03:01 | 2 | 0 |
| 04:08~03:04 | 1 | 0 |
| 04:10~03:02 | 1 | 0 |
| 04:13~03:02 | 3 | 0 |
| 04:41~04:02 | 0 | 1 |
| 07:01~02:01 | 3 | 2 |
| 07:01~02:05 | 0 | 1 |
| 07:01~03:03 | 1 | 1 |
| 08:01~04:02 | 0 | 1 |
| 08:02~03:01 | 0 | 1 |
| 08:04~03:01 | 1 | 2 |
| 08:04~03:04 | 1 | 0 |
| 08:04~05:02 | 1 | 0 |
| 09:01~02:01 | 1 | 0 |
| 09:01~03:02 | 3 | 0 |
| 09:01~03:03 | 1 | 0 |
| 10:01~02:01 | 1 | 0 |
| 11:01~03:04 | 0 | 1 |
| 11:01~06:01 | 0 | 1 |
| 11:01~06:03 | 0 | 1 |
| 11:02~02:01 | 0 | 1 |
| 11:02~03:01 | 0 | 2 |
| 11:02~03:19 | 0 | 1 |
| 11:02~06:03 | 1 | 0 |
| 11:03~03:01 | 0 | 1 |
| 11:04-03:01 | 1 | 9 |
| 11:04~06:03 | 0 | 1 |
| 11:10~03:04 | 0 | 1 |
| 12:01~03:01 | 0 | 1 |
| 12:01~05:01 | 0 | 2 |
| 12:02~03:01 | 0 | 3 |
| 12:10~03:01 | 0 | 1 |
| 13:01~03:03 | 0 | 1 |
| 13:01~05:01 | 0 | 1 |
| 13:01~06:03 | 1 | 4 |
| 13:01~06:08 | 0 | 1 |
| 13:02~06:03 | 0 | 1 |
| 13:02~06:04 | 0 | 6 |
| 13:02~06:05 | 0 | 1 |
| 13:02~06:09 | 1 | 2 |
| 13:03~03:01 | 0 | 2 |
| 13:03~06:01 | 0 | 1 |
| 14:01~05:03 | 0 | 1 |
| 14:02~03:01 | 0 | 1 |
| 14:04-05:03 | 1 | 6 |
| 14:54~05:03 | 0 | 1 |
| 15:01~05:02 | 0 | 3 |
| 15:01~06:01 | 0 | 5 |
| 15:01~06:02 | 2 | 4 |
| 15:01~06:03 | 1 | 0 |
| 15:02~05:01 | 1 | 1 |
| 15:03~06:02 | 0 | 2 |
| 15:03~06:03 | 0 | 1 |
| 15:06~05:02 | 0 | 2 |
| 15:11~06:01 | 0 | 1 |
| 16:02~06:01 | 0 | 1 |
| 16:09~05:02 | 0 | 1 |
| 16:10~05:02 | 0 | 1 |

**Supplementary material Table S5 Global linkage disequilibrium between *DRB1* and -*DQB1* loci in Patients and Controls**

| **Measure** | **T1D Patients** | **Controls** |
| --- | --- | --- |
| D’ | 0.9189 | 0.9504 |
| Wn | 0.7454 | 0.7183 |
| W*_DRB1/DQB1_* | 0.8852 | 0.8956 |
| W*_DQB1/DRB1_* | 0.7135 | 0.7131 |
